# Automated echocardiographic left ventricular dimension assessment in dogs using artificial intelligence: Development and validation

**DOI:** 10.1111/jvim.17012

**Published:** 2024-02-16

**Authors:** Catherine C. Stowell, Valeria Kallassy, Beth Lane, Jonathan Abbott, Kieran Borgeat, David Connolly, Oriol Domenech, Joanna Dukes‐McEwan, Luca Ferasin, Josefa Fernández Del Palacio, Chris Linney, Jose Novo Matos, Ilaria Spalla, Nuala Summerfield, Tommaso Vezzosi, James P. Howard, Matthew J. Shun‐Shin, Darrel P. Francis, Virginia Luis Fuentes

**Affiliations:** ^1^ National Heart and Lung Institute (NHLI) Imperial College London UK; ^2^ Clinical Science and Services Royal Veterinary College London UK; ^3^ Department of Small Animal Clinical Sciences, College of Veterinary Medicine University of Tennessee Knoxville Tennessee USA; ^4^ Department of Cardiology Eastcott Veterinary Referrals Swindon UK; ^5^ Cardiology Department Istituto Veterinario di Novara Novara Italy; ^6^ Department of Small Animal Clinical Science, School of Veterinary Science University of Liverpool Liverpool UK; ^7^ Specialist Veterinary Cardiology Consultancy Four Marks Newbury UK; ^8^ Department of Animal Medicine and Surgery University of Murcia Murcia Spain; ^9^ Cardiology Department Paragon Veterinary Referrals Wakefield UK; ^10^ Department of Veterinary Medicine University of Cambridge Cambridge UK; ^11^ Cardiology Department Ospedale Veterinario San Francesco Milan Italy; ^12^ Cardiology Service Virtual Veterinary Specialists Harrow UK; ^13^ Department of Veterinary Sciences University of Pisa Pisa Italy

**Keywords:** artificial intelligence, echocardiography, vendor‐neutral

## Abstract

**Background:**

Artificial intelligence (AI) could improve accuracy and reproducibility of echocardiographic measurements in dogs.

**Hypothesis:**

A neural network can be trained to measure echocardiographic left ventricular (LV) linear dimensions in dogs.

**Animals:**

Training dataset: 1398 frames from 461 canine echocardiograms from a single specialist center. Validation: 50 additional echocardiograms from the same center.

**Methods:**

Training dataset: a right parasternal 4‐chamber long axis frame from each study, labeled by 1 of 18 echocardiographers, marking anterior and posterior points of the septum and free wall.

**Validation Dataset:**

End‐diastolic and end‐systolic frames from 50 studies, annotated twice (blindly) by 13 experts, producing 26 measurements of each site from each frame. The neural network also made these measurements. We quantified its accuracy as the deviation from the expert consensus, using the individual‐expert deviation from consensus as context for acceptable variation. The deviation of the AI measurement away from the expert consensus was assessed on each individual frame and compared with the root‐mean‐square‐variation of the individual expert opinions away from that consensus.

**Results:**

For the septum in end‐diastole, individual expert opinions deviated by 0.12 cm from the consensus, while the AI deviated by 0.11 cm (*P* = .61). For LVD, the corresponding values were 0.20 cm for experts and 0.13 cm for AI (*P* = .65); for the free wall, experts 0.20 cm, AI 0.13 cm (*P* < .01). In end‐systole, there were no differences between individual expert and AI performances.

**Conclusions and Clinical Importance:**

An artificial intelligence network can be trained to adequately measure linear LV dimensions, with performance indistinguishable from that of experts.

**Abbreviations**
2D: 2‐dimensional
AI: artificial intelligence
ED: end diastole
ES: end systole
FW: free wall
IVS: interventricular septum
LV: left ventricle
LVD: left ventricular diameter

## 1 INTRODUCTION

Artificial intelligence (AI) techniques have been successfully applied to automate human‐led diagnostic imaging analysis tasks. Within veterinary medicine, this research has so far focused on radiology rather than echocardiography.^1^, ^2^ Of the 40 peer‐reviewed studies since 2015, none used AI to evaluate echocardiographic images.^3^, ^4^

AI has potential to contribute automatic assistance in the analysis of echocardiographic images.^5^ These potential applications could result in reduced scan times, lower the skill‐floor necessary to perform this diagnostic, and improve efficiency of repeat measurements, recommended as a standard of good practice.

However, AI should be assessed objectively, as is the case for any diagnostic test or expert opinion. Our study seeks to provide this data in an effort to evaluate the technology for eventual deployment in mainstream practice.

Measurement of left ventricular (LV) wall thickness and internal diameter are key measurements. There are not yet published guidelines on how these measurements should be performed, and different veterinarians use different views, such as 2D or M‐mode, long‐axis or short‐axis.

In canine echocardiography, the right parasternal 4‐chamber view is often the first view acquired and provides an immediate impression of size and function. In this study, we develop and validate an automated system for measuring wall thickness and left ventricular internal diameter.

For human patients, echocardiography is generally performed in a laboratory where many practitioners perform scans, using a standard protocol. Laboratories typically perform audits assessing adherence to acquisition protocols, and reproducibility studies to assess adherence to measurement protocols.^6^ Veterinary echocardiography is generally performed at lower volumes and often by individuals working alone.

To formulate a standard against which the developed system could be tested, our study therefore had to capture opinions from a range of experts that could be used as a consensus.

In this article, the term “validation” is used exclusively to refer to final evaluation of the completed AI model on unseen data. This is in line with conventional use of this term in medical statistics, and the TRIPOD‐ML guideline.^7^ AI literature instead uses the word “test” to refer to this final evaluation, because it uses “validation” to refer to the internal progress monitoring of model performance during the development and training process.

During the training process of an AI, typically a small number of dedicated experts label images, which are then used for training the network. Inevitably, the performance of the AI on interpreting those images it has already seen will be high. A more subtle problem, however, is that if the experts providing the training data have certain biases, the AI will simply reflect those biases. To properly test the AI's performance, therefore, the validation must not only (a) use images that have never been used during training, but also (b) use a sufficiently broad range of experts that any systematic biases the AI has learned from the trainers will be exposed.

## 2 METHODS

Under the categorization laid out by Campbell et al, this study falls into the “development and validation” stage, rather than a randomized trial of an intervention.^8^ Of the 2 guidelines cited, only TRIPOD‐ML has been published.^7^ The TRIPOD‐ML checklist is submitted as Appendix S2.

The study consisted of 5 phases. First, training images were systematically extracted from a clinical database. Second, a team of echocardiographers labeled points on images. Third, the AI was trained to replicate the behavior of the echocardiographers on those training images. Fourth, a fresh, non‐overlapping set of 100 images was extracted from the clinical database. Finally, a broad team of 13 experts each reviewed all of the 100 images, twice. We used these opinions to measure the AI's performance on each image separately.

For each image, we defined the gold standard as the mean of the expert opinions on that particular image. For that image, the deviation of the AI away from that value was considered the AI error. In parallel, the deviation of the individual experts away from that same expert consensus could be considered an individual‐expert error. The size of the AI error on that image could then be examined in the context of the spectrum of errors from individual experts for that image. If, across all images, the AI errors were similar in size to the individual‐expert errors, the AI might be considered to be performing adequately.

### 2.1 Extraction of the training images

The training images were from all echocardiography examinations of dogs recorded by the cardiology service at the Royal Veterinary College between 1 January 2018 and 31 December 2019. The process was systematic, with no exclusions on the basis of image quality or breed. The only ineligible cases were those with no right parasternal 4‐chamber view. From each right parasternal 4‐chamber video, random frames were extracted, irrespective of timing within the cardiac cycle. This was so that the AI would have exposure to the broadest range of appearances of the heart throughout the cardiac cycle and might in future have the potential to track points continuously, even though the current aim was only to make measurements at the clinically relevant timepoints. Several thousand images were extracted, to be more than sufficient for each echocardiographer to label as many as they desired.

### 2.2 Echocardiographer labeling of training images

**FIGURE 1:**
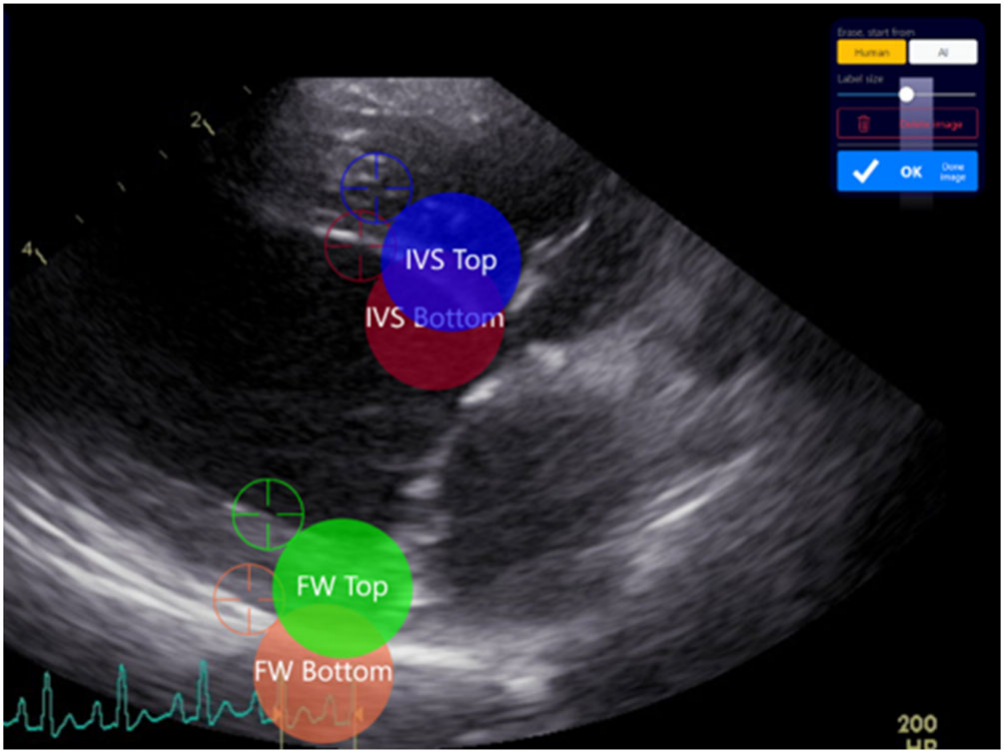
The Unity application interface provides a web‐based interface to annotate medical images. The 4 adjustable electronic calipers are highlighted as colored circles with their names and associated target icons for their exact location. Key points can be labeled either using a touchscreen interface or a mouse.

Veterinary echocardiographers were invited to participate. Using the Unity Imaging platform (Figure 1), they were asked to label the anterior and posterior points of the septum and free wall. Each echocardiographer was required to label at least 70 images but was allowed to label more if they wished. This minimum of 70 was chosen from previous experience and discussion with veterinary experts as an achievable level of commitment to require.^9^ No upper limit was placed, so that echocardiographers who were more able to commit time could contribute more.

Our collaboration of 13 trained veterinary echocardiographers, and 5 trainees (veterinary students or veterinarians) annotated a total of 1914 image frames from 461 canine echocardiographic studies. Each frame was labeled in 4 locations using electronic calipers (see Figure 1), corresponding to the anterior and posterior borders of the interventricular septum and the left ventricular (LV) free wall, and yielding the left ventricular (LV) internal diameter and septal and free wall thicknesses. Instructions were given to avoid deleting any image unless the image quality was so poor as to make it impossible to place a single measurement point.

Unity Imaging is an online application and has been previously described.^9^, ^10^ Unity is used to display and obtain annotations from medical images in an engaging, intuitive, and user‐friendly way. When a new project is created, the key measurement points (or landmarks) are specified, between which measurements are made, as illustrated in Figure 1.

### 2.3 Training the AI to replicate echocardiographer labels

**FIGURE 2:**
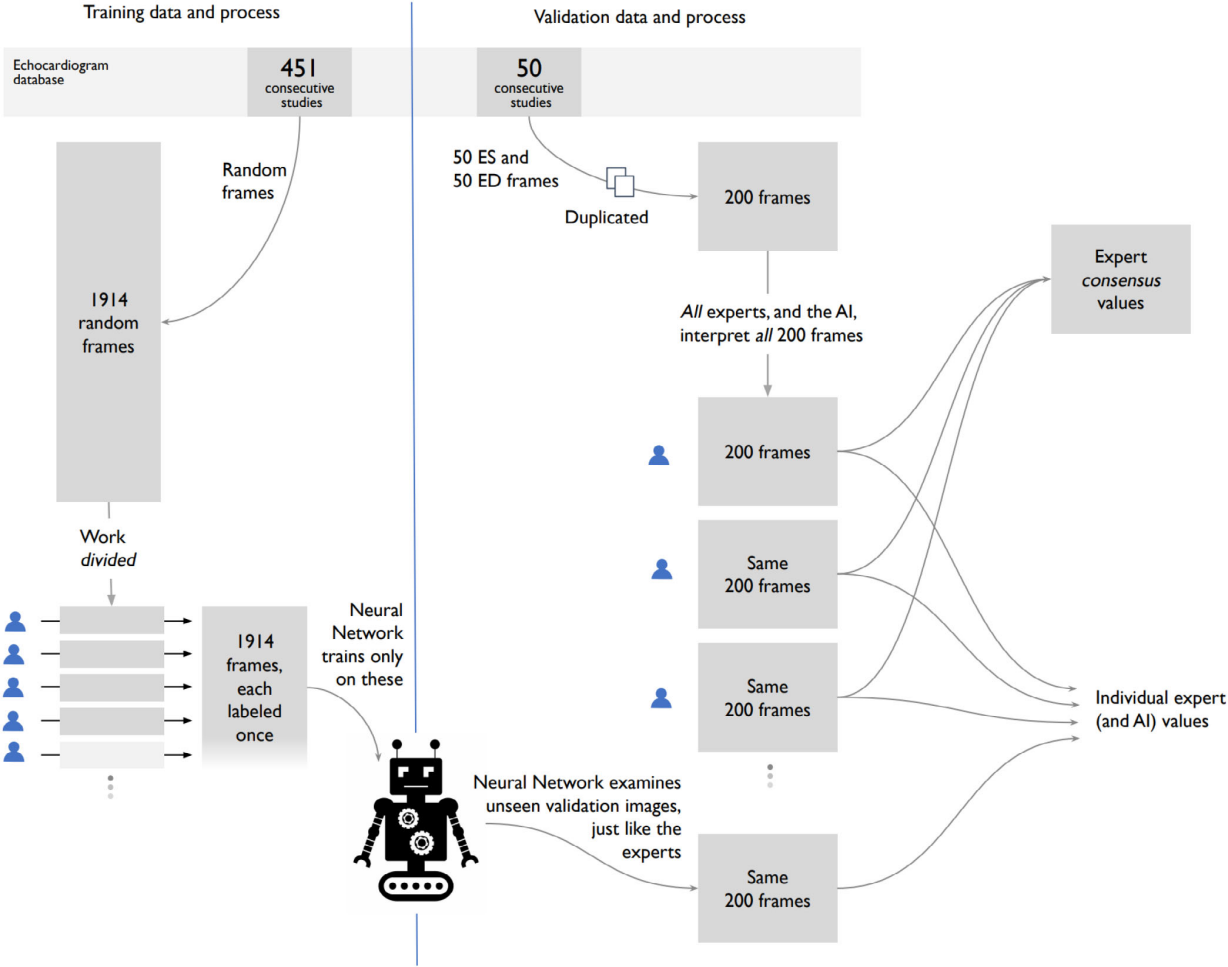
There were 2 echocardiographic datasets. Each of the 1914 frames in the *training dataset* was labeled by 1 of 18 team members. The external *validation dataset* comprises 100 frames, each labeled twice by each of the 13 veterinary experts, providing 26 labels for each frame.

We created 2 canine echocardiographic datasets (Figure 2) of images of the right parasternal 4‐chamber view. They came from distinct calendar periods in the clinical echocardiogram database.

The *training dataset* of 1914 frames was used for training and monitoring of the AI network. Each frame was labeled once, by 1 of 13 veterinary experts (72% of all frames), or 1 of 5 trainees (28% of all frames).

The external *validation dataset* comprised 100 frames: the manually selected end‐diastole (ED) and end‐systole (ES) images from 50 separate echocardiographic scans. Each of the 100 frames was labeled twice (blindly) by each of the 13 veterinary experts, providing 26 labels for each frame. The validation dataset is referred to as “external,” meaning none of these images were used in the training phase.

## 3 VALIDATION DATASET

The external validation dataset comprises 100 end‐diastolic (ED) and end‐systolic (ES) images from 50 consecutive canine echocardiography studies from the Royal Veterinary College during 2018. The validation dataset was from a distinct non‐overlapping calendar period.

Images were presented in random order, and each was labeled twice by the 13 experts. A dataset was formed of 200 labeled frames (100 images) per expert. Each expert was blinded to their own and others' labeling. It was not possible for the experts to delete images with suboptimal image quality from the dataset: every image had to be labeled.

This sample size was set in conformance with our previous experience as providing a representative range of images, which would cover a spectrum of image qualities. The 200 frames, each labeled 26 times, led to 5200 sets of 3 measurements.

## 4 ARTIFICIAL INTELLIGENCE NETWORK TRAINING

**FIGURE 3:**
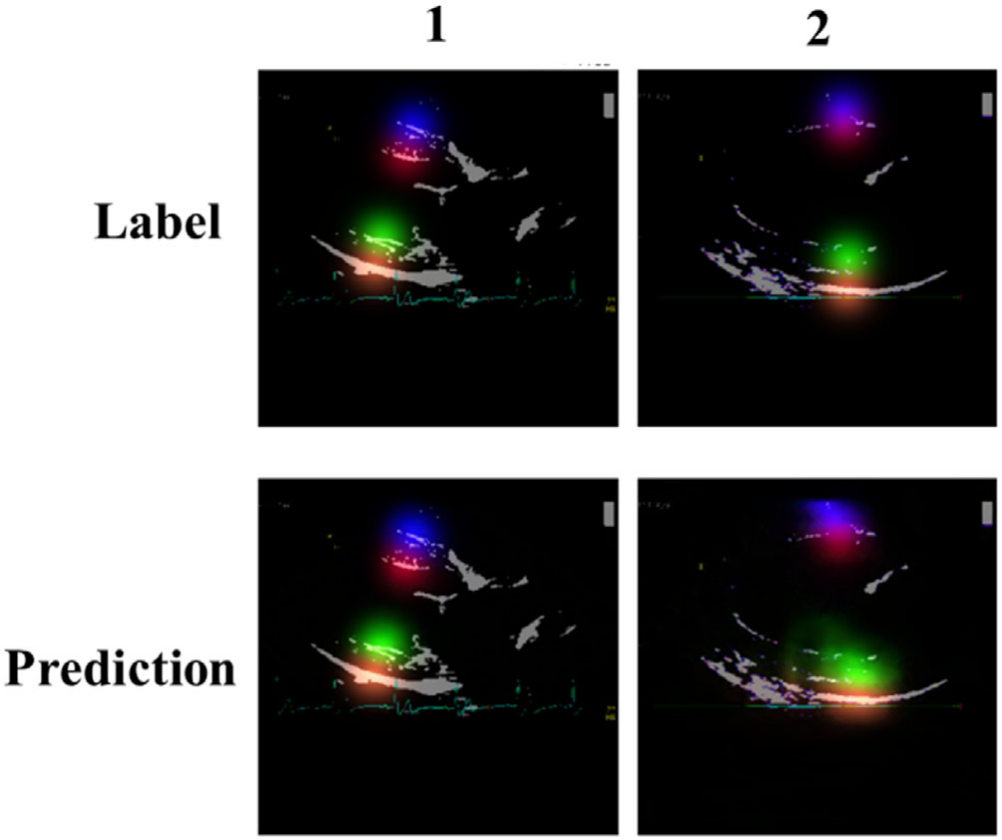
Examples of Gaussian heatmaps for expert annotations (top) and network predictions (bottom). It can be observed that the image in column 1 generates more favorable predictions than those in column 2; possibly attributed to lower image clarity.

An artificial neural network was trained to infer 4 key points (as illustrated in Figure 1). Network predictions are in the form of a heatmap for each key point, with a Gaussian distribution SD of 15 pixels. Cartesian coordinates for each key point were derived by locating the local maxima from the corresponding predicted heatmap, examples of which can be found in Figure 3.

**FIGURE 4:**
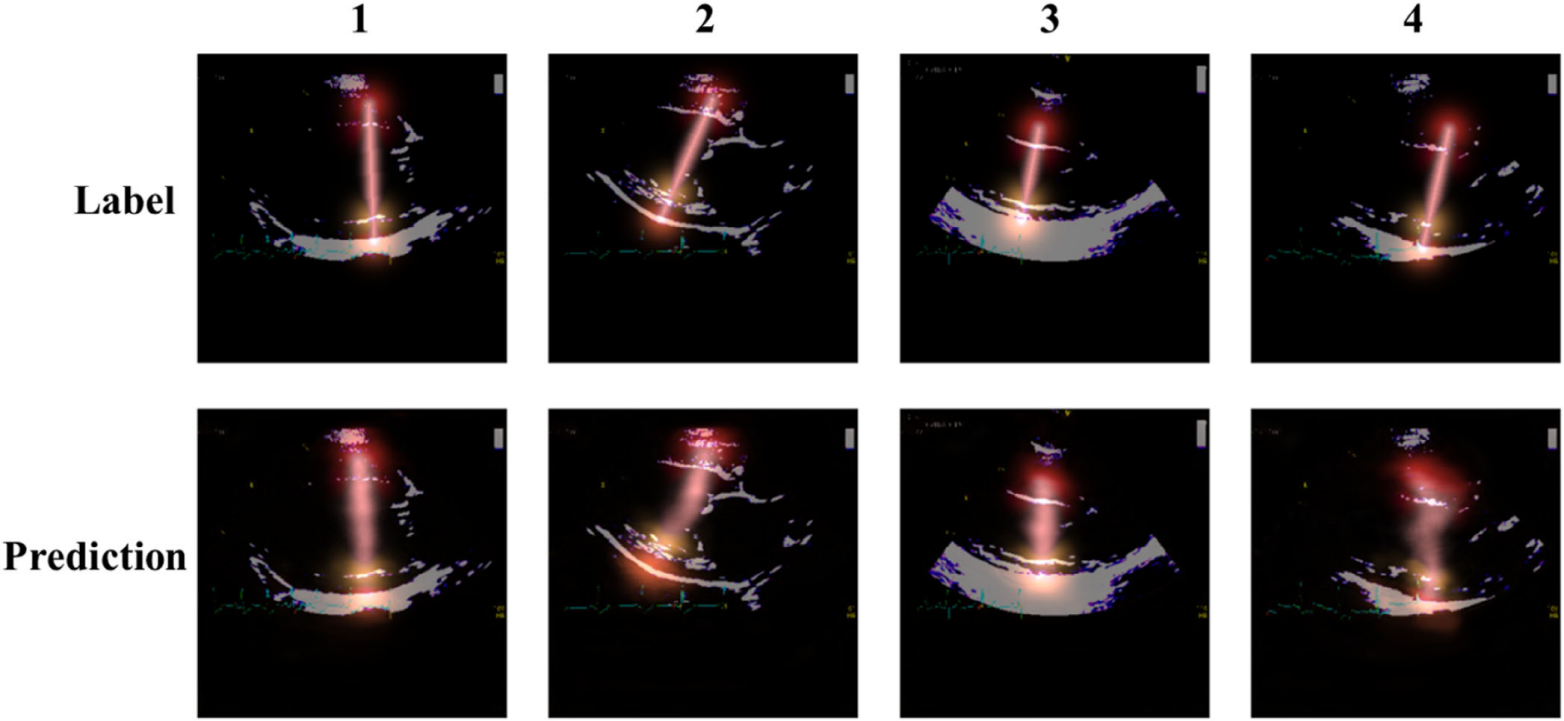
Examples of a biological constraint in the form of a pseudo line, connecting all 4 key points in a straight line. The top row shows annotations and the bottom are predictions generated during the training phase. Column 1 represents an accurate prediction, whereas 4 is less clear. This could be attributed to lower image quality for this particular example.

The well‐established U‐Net architecture was used for the network backbone, with semantic segmentation masks produced for each training image.^11^ The code and detailed methods are given at https://data.unityimaging.net. All images were resized to a uniform dimension of 320 × 320 pixels, with 3 color channels. Of the entire dataset (1914 images), 80% were used for network training while 20% were retained for testing. Random, on‐the‐fly augmentation, including affine transformation and random gamma changes, was applied. Additionally, in an effort to ensure consistency of alignment for all 4 measurement points, a biological constraint was applied in the form of a pseudo line during training, as observed in Figure 4.

The network was trained for a total of 200 epochs, distributed across a cluster of 4 Nvidia GeForce RTX 3090 GPUs and using Pytorch version 1.13 and Python version 3.9. The initial learning rate was 0.001, using the TAdam optimizer and mean squared error loss function. The learning rate was reduced by a factor of 5 each time the loss on the testing dataset plateaued for 20 consecutive epochs. If an expert was unable to localize the key point on an image (eg, due to very poor image quality), the training process did not train on that key point of that image (by weighting the loss function to 0).

Once the network training was completed, performance was then analyzed against predictions made on the external validation dataset (comprising 100 images). A comprehensive overview of AI concepts and theory, along with a review of the current literature in the field can be found in the references.^2^, ^3^

## 5 VALIDATION AGAINST EXPERT CONSENSUS

In this study the 26 expert labels, on each of the 100 validation frames, were used in 2 ways. First, the mean of the 26 values was used to define the reference standard for that measurement on that frame. Second, the spectrum of variation between different expert opinions was used to define the acceptable range of expert performance, against which the AI should be judged.

For each measurement on each image, the consensus value was defined as the mean of the 26 expert values (13 experts measuring twice). Using this, the deviation of each expert from this consensus was calculated, for example, +2 mm if that expert's value was greater than the consensus, and for example, −3 mm if that expert's value was smaller than the consensus. While the mean of these deviations will always be 0, the SD (or root‐mean‐square) of these individual deviations is a useful index of dispersion.

This was done for each measured structure (IVS, LVD, FW), separately for the end‐systolic and end‐diastolic frame.

We then similarly calculated for each image and measurement site, the deviation between the AI assessment and the consensus of the experts.

Finally, we tested whether the size of the deviations of the AI assessments (away from the consensus of experts) was different from the size of the deviations of the individual expert assessments (away from that same consensus of experts). We did this with the Levene test using the “Levene” package in SciPy 1.10.1, using Python 3.10. We also tested whether the AI assessments showed a mean bias away from the consensus of experts, using the paired *t* test.

Routine descriptive statistics, such as mean, median and SD, were calculated using Python 3.10.

## 6 RESULTS

The study cohort included purebred and crossbreed dogs, with a mean weight of 18.6 kg, ranging from 0.72 kg (neonatal Pug) to 108 kg (Saint Bernard), as shown in Appendix S1.

The 100 frames of the external validation dataset had more elaborate measurements, in 2 regards: each study contributed 2 frames (ED and ES), and each were analyzed 26 times (twice, by each of the 13 experts). For each measurement, the reference standard for was defined to be the mean of the 26 judgments. The characteristics of these 50 echo studies are shown in Table 1.

**TABLE 1:** Deviations from expert consensus, of the individual experts and of the AI (end‐diastolic frames).

End‐diastole deviations from consensus (cm)							*P*_(Comparison of SDs)_	*P*_(Comparison of means)_
Measurement	Individual expert deviations from expert consensus			AI deviations from expert consensus				
	Mean	SD	Median	Mean	SD	Median		
IVS	0.00	0.12	0.00	0.05	0.11	0.06	.61	.001
LVD	0.00	0.16	0.00	0.05	0.13	0.06	.65	.02
FW	0.00	0.10	0.00	0.01	0.13	0.00	.004	.36

Table 1 shows how much the individual expert opinions varied from the reference standard, for the corresponding case. This is shown as a root‐mean‐squared deviation (equivalent to the SD). The table also shows how much the AI measurement varied from the reference standard, again, as a root‐mean‐squared deviation.

For most of the comparisons, there was no significant difference between the size of the deviations, between AI and experts. For IVS and LVD, it was smaller for the AI, and for FW it was the same or larger for the AI (Tables 1 and 2).

**TABLE 2:** Deviations from expert consensus, of the individual experts and of the AI. End‐systolic frames.

End‐systole deviations from consensus (cm)							*P*_(Comparison of SDs)_	*P*_(Comparison of means)_
Measurement	Individual expert deviations from expert consensus			AI deviations from expert consensus				
	Mean	SD	Median	Mean	SD	Median		
IVS	0.00	0.16	0.01	0.05	0.16	0.05	.84	.03
LVD	0.00	0.25	0.00	0.03	0.25	0.02	.96	.39
FW	0.00	0.17	0.00	0.01	0.14	0.03	.44	.54

The rightmost column shows the test of bias between the AI measurements and the expert consensus. There were small but statistically significant positive biases for diastolic IVS (mean bias = +0.05 cm, SD 0.16 cm, *P* < .01); diastolic LVD (mean bias = +0.05 cm, SD 0.13 cm, *P* = .02) and systolic IVS (mean bias = +0.05 cm, SD 0.16 cm, *P* = .03), compared with the experts.

**FIGURE 5:**
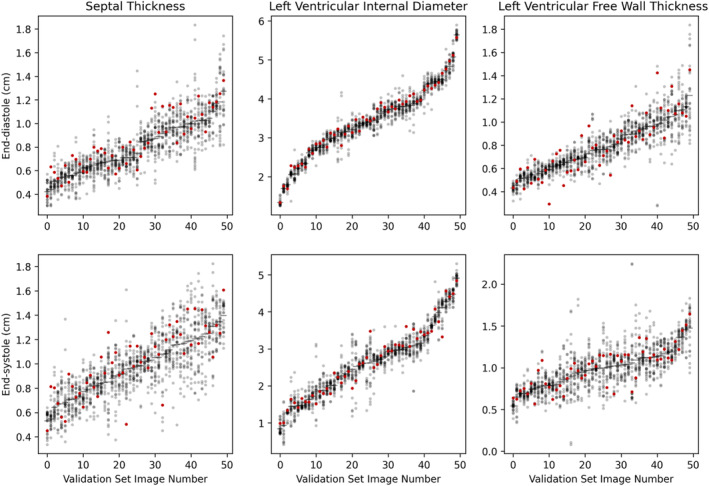
Relationship of AI value (red) to the 26 expert values (gray), for each of 50 end‐systolic and 50 end‐diastolic frames. The frames are arranged by the consensus measurement, with the smallest first. The 3 panels display IVS, LVD and FW, respectively.

To gain a visual impression of the performance of the AI against the performance of experts acting individually, we plotted, for each case, the 26 expert measurements as a series of gray points, with the AI measurement as a red point. For visual convenience, we ordered the cases in each plot by the expert consensus measurement. As can be seen (Figure 5), the AI's performance is similar to that of the experts.

**FIGURE 6:**
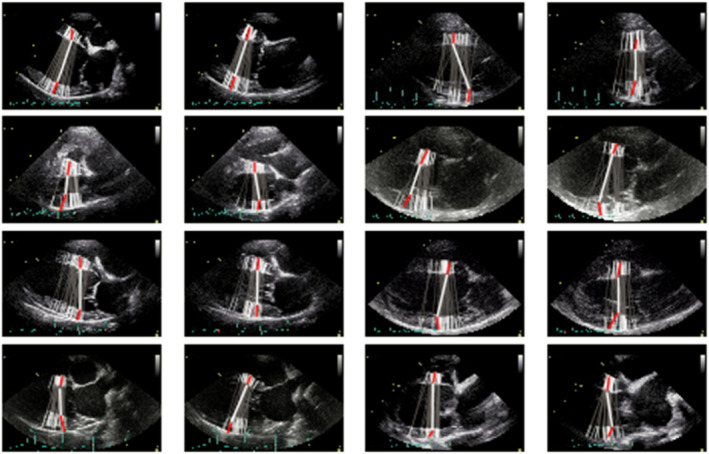
A selection of images from the external validation dataset. Expert labels are represented by the gray lines with white tips, with the network prediction bold white with red tips.

Generally, the experts agreed on wall thickness (vertical position), but not on the location for measurement within the septum and free wall (horizontal position). Each expert opinion on a random selection of validation images is shown in Figure 6, as are the corresponding AI predictions (a thicker line in white and red). We note that the AI tends to choose a longitudinal position that is near the center of the range of positions chosen by different experts (Figure 1). In no case was the AI's choice more apical or more basal than all the expert opinions. In the third panel, the AI is taking an unacceptably diagonal path across the left ventricle. In the fourth, it might be mis‐measuring the thickness of the left ventricle, although there is considerable variation among individual experts, suggesting that the correct measurement is debatable. In the 13th panel, the posterior wall is overestimated, and in the 15th, underestimated.

**FIGURE 7:**
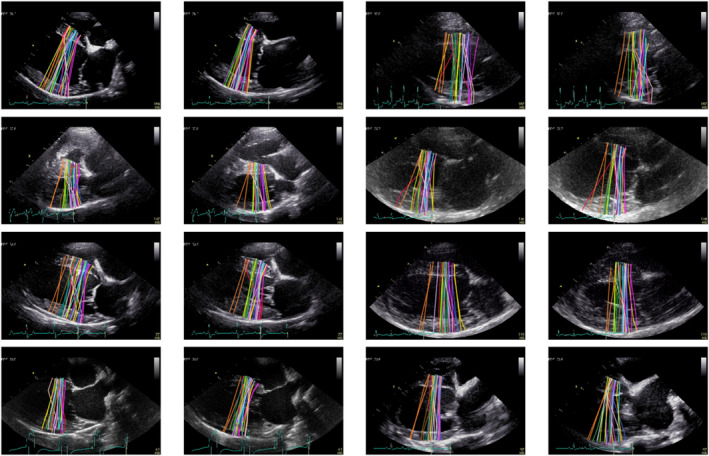
Examples of expert annotations for the external validation dataset (100 images). Each of the 13 human experts annotated each image twice, therefore a random color assignment is applied for the purpose of visualizing those labels made by individuals.

Incidentally, it was noticed that some experts tended to choose positions more apical or basal than other colleagues. This can be seen in Figure 7, which no longer attempts to depict the position of individual fiducial points, but rather, uses color solely to separate the individual experts. The 2 opinions of an expert are colored identically and consistently across the cases.

The curated dataset and corresponding expert annotations, used for training and validation of the AI network, along with the associated code files, have been made freely available for the benefit of the research community. For access, please visit the project website.^10^ Ethical approval was obtained from the Social Science Ethics Review Board at the Royal Veterinary College (URN: SR2022‐0060).

## 7 DISCUSSION

This study shows that expert clinicians working in different countries can collaborate to train a neural network to perform core echocardiographic measurements. Second, it shows how the performance of this network can be judged in a separate study of validation cases, against the consensus of multiple experts. Third, the resulting system performs similarly to human experts but is not perfect: we show examples of errors made, because these will stimulate further developments. A process such as this could be useful in the construction of future clinical guidelines, and validation data assembled in this way might be useful in training future clinicians.

Online collaboration enabled individuals who could otherwise not be easily brought together, to cooperate on a shared project. Expert echocardiographers in different countries were able to collaborate effectively in the training and validation process.

Our validation data was demanding on the experts, because each of the 13 experts had to perform 200 image labelings, but this approach provides 2 benefits.

First, the consensus of expert opinion becomes far more consistent when a large number of experts are combined, and further enhanced when each expert makes the assessment more than once. This is important because, if the reference standard contains noise, that will put a floor on how closely the AI can match the reference standard. The greater the number of expert opinions combined, the more reliable the consensus, and therefore the greater the opportunity to detect high performance from the AI.

Second, it has been unclear how strong a performance should be demanded from an AI measurement system. Having systematically collected expert opinions provides a trustworthy scale against which the performance of the AI can be judged. One might prespecify that it needs to agree with the consensus at least as well as the worst‐performing expert, or no worse than the median‐performing expert, or any other such target. Setting standards in this way automatically allows the AI to be held to a stricter absolute standard on easy images, and a looser standard on difficult images where experts themselves disagree.

It is important to note there is currently no consensus in veterinary echocardiography on how to measure left ventricular wall thickness or internal diameter, and this might be a reason why there was variation even among experts on sites chosen for measurement. The methodology used in this study could be useful for capturing expert opinion, and establishing a consensus, for the creation of future guidelines. Equally, repeating this study once guidelines are published could yield different results.

The utility of a training set of images and expert labels is so great that it might be considered a good way for specialist societies to guide learners on how measurements should be made. This approach avoids the difficulty of articulating annotation points unambiguously in words. If the guidance is the form of multiple independent expert opinions, it also has the advantage of demonstrating to learners where variation is acceptable, and where it is not. Finally, such datasets have the potential to be used not only by trainees, but also by AI research workers aiming to deliver systems that meet that standard.

We chose the right parasternal 4 chamber view, although most vets do not routinely measure wall thickness or left ventricular internal diameter from this view, with the short axis being preferred (whether in 2D or M‐mode). It could potentially be that both intra‐ and inter‐operator measurement variability would be lower in more familiar views. However, this does not change the overall conclusions of this study, as the challenges faced by human experts applied equally to the AI that was originally trained by them.

Although the AIs performed similarly to individual experts, in terms of its deviation away from the expert consensus, it did show a small bias toward reporting larger measurements, by about 0.5 mm, which was statistically significant when pooled across 50 echocardiograms. Although this does not mean the AI is invalid (since this level of deviation from expert consensus is acceptable for individual experts), it does underline the need for careful testing of automated systems in realistic cohorts of images, with an expert panel to form a reference standard.

Expert echocardiographers working at different locations can collaborate online to train an AI to perform as well as they do. We recommend that using a multi‐expert consensus is best practice for defining a reference standard against which to test an AI.

Systems using AI have the potential to streamline the workflow of experts, and to improve uniformity of practice standards. For such systems to become routinely adopted, their full methodology should be publicly accessible and amenable to improvement, and their validation process should be open and explicit, under the aegis of named experts.

## CONFLICT OF INTEREST DECLARATION

Authors declare no conflict of interest.

## OFF‐LABEL ANTIMICROBIAL DECLARATION

Authors declare no off‐label use of antimicrobials.

## INSTITUTIONAL ANIMAL CARE AND USE COMMITTEE (IACUC) OR OTHER APPROVAL DECLARATION

Approved by the Social Science Ethics Review Board of the Royal Veterinary College (URN: SR2022‐0060).

## HUMAN ETHICS APPROVAL DECLARATION

Authors declare human ethics approval was not needed for this study.

## Supporting information


**Appendix S1.** Training dataset.

**Appendix S2.** TRIPOD checklist: Prediction model development and validation.
