# Supplementary material for: Automated echocardiographic left ventricular dimension assessment in dogs using artificial intelligence: Development and validation
Source: J Vet Intern Med. 2024 Feb 16;38(2):922–30. doi: 10.1111/jvim.17012 (PMC10937473; doi:10.1111/jvim.17012)
Supplement: Supplementary file 1 — Appendix S1. Training dataset. [file JVIM-38-922-s002.pdf]

Appendix 1 - Training Dataset

The frames from the 1914 images of the training dataset were taken from a variety of timepoints in the cardiac cycle, and not solely end-systolic and end-diastolic. This was to maximize the exposure of the neural network to the full range of behavior of the ventricle.

Each frame was single-labeled by a trainer, specifically for this research project. Routine clinical measurements were not used, because this study did not have access to clinical measurements and because this study wished to train on frames throughout the cardiac cycle.

Appendix 1, Table 1

We tabulated the breed and weight of all dogs receiving a departmental transthoracic echocardiogram in 2018.

Breed	Number in Study	Avg. Weight (kg)
Affenpinscher	1	4.0
Airedale Terrier	1	32.0
Alaskan Malamute	1	63.5
Basset Hound	3	26.6
Beagle	1	23.5
Bearded Collie	1	12.8
Bernese Mountain Dog	1	55.5
Bichon Frise	7	7.1
Border Collie	7	18.4
Boston Terrier	2	8.0
Bouvier De Ardennes	1	38.1
Boxer	17	30.4
Bruxellois Griffon	1	5.3
Bulldog	2	20.6

Cairn Terrier	3	7.7
Cavalier King Charles Spaniel	39	10.4
Chesapeake Bay Retriever	1	29.8
Chihuahua	23	4.4
Cocker Spaniel	21	13.8
Collie	1	29.0
Coton De Tulear	1	5.9
Crossbreed	74	15.0
Czechoslovakian Wolfdog	1	28.5
Dalmatian	3	27.7
Doberman Pinscher	8	35.0
Dogue De Bordeaux	5	61.0
English Bulldog	2	24.3
English Pointer	1	25.4
English Setter	1	29.9
English Springer Spaniel	5	24.6
English Staffordshire Bull Terrier	1	22.2
Flat-Coated Retriever	1	37.6
Fox Terrier	1	8.3

French Bulldog	7	11.7
German Shepherd Dog	8	38.6
Golden Retriever	11	30.2
Great Dane	2	61.3
Greyhound	5	31.0
Hungarian Vizsla	3	27.7
Husky	2	30.0
Irish Terrier	1	20.0
Jack Russel Terrier	13	6.7
Japanese Spitz	2	10.9
King Charles Spaniel	1	14.7
Labrador	28	29.4
Leonberger	2	53.1
Lhasa Apso	2	6.4
Lurcher	2	21.8
Maltese	7	3.7
Miniature Dachshund	6	6.1
Miniature Pinscher	2	2.7
Miniature Schnauzer	7	10.8
Neapolitan Mastiff	1	68.6

Newfoundland	6	57.7
Norfolk Terrier	1	7.0
Old English Sheepdog	1	38.0
Parson Russell Terrier	2	9.3
Patterdale Terrier	2	9.3
Pekingese	2	6.0
Polish Lowland Sheepdog	1	17.6
Pomeranian	4	4.4
Pug	2	3.2
Rhodesian Ridgeback	1	48.3
Rottweiler	3	52.8
Rough Collie	1	16.8
Saint Bernard	1	108.0
Schnauzer	1	10.5
Shar-Pei	1	22.0
Shih Tzu	5	8.0
Soft-Coated Wheaten Terrier	2	18.0
Spanish Water Dog	1	14.3
Springer Spaniel	3	14.5
Staffordshire Bull Terrier	7	18.4

Standard Dachshund	3	17.2
Standard Poodle	2	26.5
Tibetan Terrier	3	10.0
Toy Poodle	2	6.4
Trailhound	1	23.0
Weimaraner	3	29.2
Welsh Border Collie	1	20.2
West Highland White Terrier	6	9.1
Whippet	3	11.3
Wire-Haired Fox Terrier	2	5.4
Yorkshire Terrier	11	6.3
